# Choroid plexus tumours on MRI: similarities and distinctions in different grades

**DOI:** 10.1186/s40644-019-0200-1

**Published:** 2019-03-20

**Authors:** Huan Lin, Xi Leng, Chun-hong Qin, Yong-xing Du, Wen-sheng Wang, Shi-jun Qiu

**Affiliations:** 1 Department of Radiology, Zhujiang Hospital of Southern Medical University, No. 253, Gong Ye Da Dao Zhong, Guangzhou, 510280 People’s Republic of China; 2 Medical Imaging Center, the First Affiliated Hospital of Guangzhou University of Traditional Chinese Medicine, No. 16, Ji Chang Lu, Guangzhou, 510405 People’s Republic of China; 3 Department of Radiology, Shunde Hospital of Southern Medical University, Foshan, People’s Republic of China; 4 Medical Imaging Center, Guangdong 999 Brain Hospital, Guangzhou, People’s Republic of China

**Keywords:** Choroid plexus tumour, Choroid plexus papilloma, Atypical choroid plexus papilloma, Choroid plexus carcinoma, Magnetic resonance imaging, Pathological grade

## Abstract

**Background:**

The therapeutic planning varies for different grades of choroid plexus tumours (CPTs). The aim of this study was to define the similarities and distinctions among MRIs for different grades of CPTs, providing more guidance for clinical decisions.

**Methods:**

We reviewed the MRI findings in 35 patients with CPT verified by surgical pathology, including 18 choroid plexus papillomas (CPPs, grade I), 11 atypical choroid plexus papillomas (aCPPs, grade II), and 6 choroid plexus carcinomas (CPCs, grade III). Nonparametric testing based on ranks was performed to evaluate the association of pathological grade with MRI findings.

**Results:**

Among the 35 CPTs, 29 were located in the ventricular system. The tumours were generally slightly hypo- or isointense on T1WI, slightly hyper- or isointense on T2WI, and moderately or strongly enhanced in post-contrast imaging. Twenty cases were accompanied by hydrocephalus. The median tumour longest diameters of CPPs, aCPPs, and CPCs were 28.6, 44.6, and 60.6 mm, respectively. Four cases were purely cystic, 6 were papillary, 10 were lobulated, and 2 were irregular. Three cases had necrosis. The median oedema diameters of CPPs, aCPPs, and CPCs were 0, 0, and 24.1 mm, respectively. The grades of CPTs were statistically associated with tumour longest diameter (*r*_*s*_ = 0.68, *P* < 0.001), internal morphology (*χ*^2^ = 10.32, *P* = 0.016), necrosis (*Z* = 2.27, *P* = 0.023), and oedema diameter (*r*_*s*_ = 0.72, *P* < 0.001).

**Conclusion:**

CPTs typically appeared as intraventricular papillary or lobulated lesions, often accompanied by hydrocephalus. Larger tumour, irregular or fuzzy internal morphology, presentation of necrosis and wide-ranging peritumoural oedema might increase the likelihood of malignancy.

## Background

Choroid plexus tumours (CPTs) are rare neoplasms derived from choroid plexus epithelium [1], representing 0.3%~ 0.6% of all intracranial tumours [2]. According to the latest WHO classification system in 2016 [3], CPTs can be classified into 3 subtypes: choroid plexus papilloma (CPP, grade I), atypical choroid plexus papilloma (aCPP, grade II), and choroid plexus carcinoma (CPC, grade III). Among all CPTs, approximately 80% were CPPs, 15% were aCPPs, and less than 5% were CPCs [4, 5]. aCPP was a new subtype introduced in 2007 [1] and is recognized as a neoplasm with intermediate histology, with more aggressive biologic behaviour, earlier metastasis and higher recurrence rates than CPPs [6].

Although surgical resection has remained the standard of care for each grade of CPTs [7], pretreatment imaging diagnosis is essential for surgical planning and therapeutic decisions [2, 7]. However, as rare neoplasms, most previous studies about CPTs have been case reports, and almost all studies lacked hypothesis tests on the relationship between pathological grades and MRI findings. Furthermore, some studies held that it was difficult to distinguish the grade of CPTs only with conventional MRI [8]. In this study, we reviewed the MRI findings of 35 CPTs with the goal of defining the MRI similarities and distinctions among grades. This would help us to obtain more information from conventional MRI and could provide more guidance for clinical decisions.

## Methods

### Clinical and pathological data

All data were collected from Zhujiang Hospital of Southern Medical University and Guangdong 999 Brain Hospital from 2006 to 2016. We performed a retrospective analysis on 35 patients (18 males, 17 females, median age 10 years with a range of 2 months to 59 years) with CPT, including 18 CPPs (grade I), 11 aCPPs (grade II) and 6 CPCs (grade III). The clinical symptoms and duration of symptoms of all patients were recorded. All patients received pretreatment MRI scanning and gross resection. The pathological diagnosis was established on the basis of histologic and immunohistochemical evidence. The cases without definite grades in pathological reports were regraded by an experienced pathologist according to the latest WHO classification system.

### MRI scan and analysis

All of the 35 patients with CPT had received pretreatment MR plain and contrast enhancement examinations. The MR scans were performed on either a 1 .5T or 3 .0T MR scanner. Plain scan was performed using axial T1-weighted, T2-weighted, FLAIR and sagittal T1-weighted sequences. Gd-DTPA was used as the contrast agent (0.2 mmol/kg). Post-contrast scan was performed using axial, sagittal and coronal T1-weighted sequences.

The MR images were analysed by at least two radiologists blinded to pathological diagnosis, and decisions were reached by consensus. The following imaging features were evaluated: tumour location (intraventricular, extraventricular), tumour longest diameter, internal morphology, signal intensity on T1WI (slightly hypo-, isointense compared to white matter) and T2WI (slightly hyper-, hypo-, isointense compared to white matter), degree of enhancement (moderate, strong), peritumoural oedema diameter, degree of hydrocephalus (none, mild, severe), whether there was haemorrhage (no, yes), necrosis (no, yes), and metastases (no, yes).

Tumour longest diameter was the largest measurement of the solid part of the tumour (excluding cysts) in post-contrast imaging. For the 4 cases without MRI-visible solid lesions, the longest diameter was recorded as 0 mm.

**Fig. 1:**
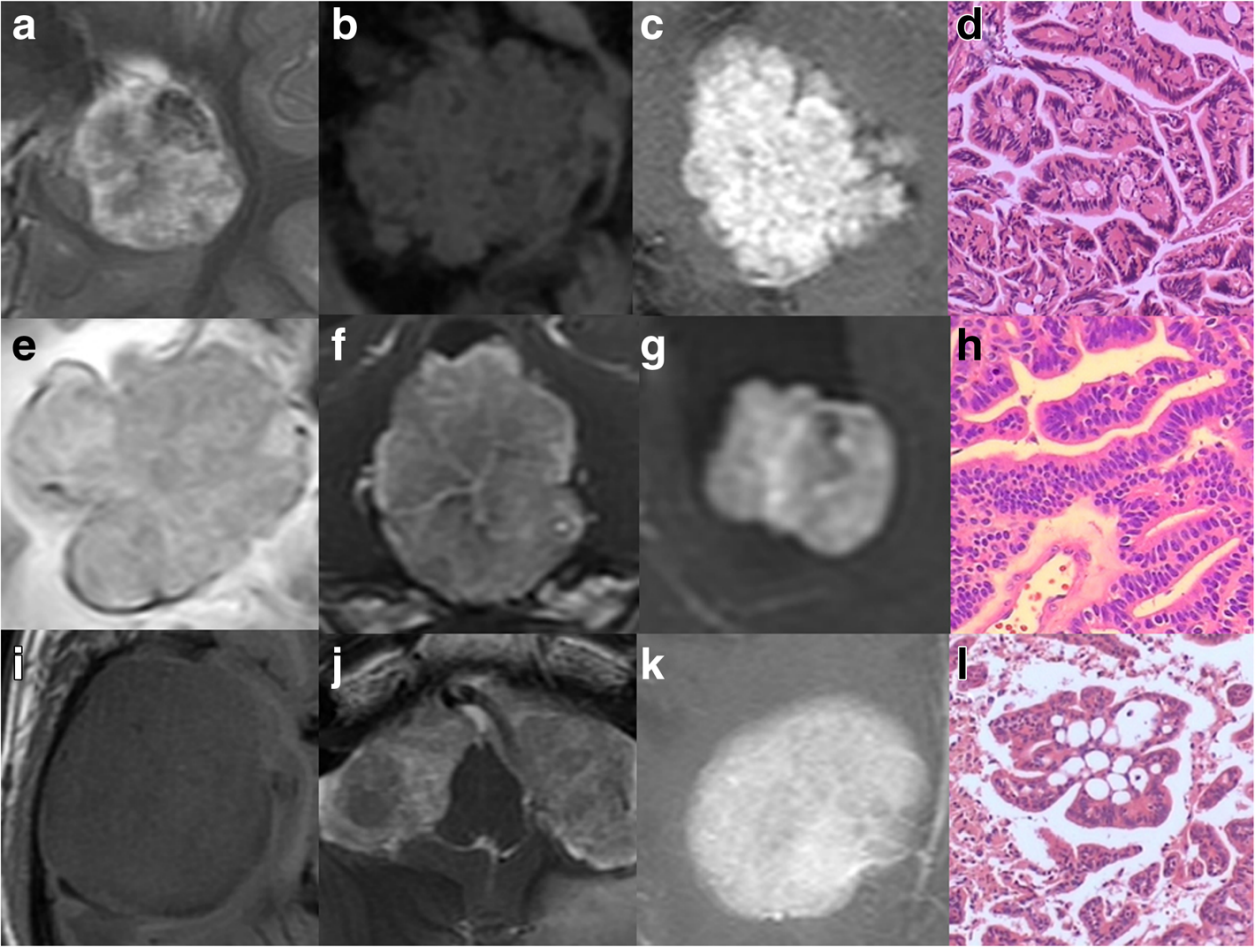
The CPTs with typical papillary/lobulated/irregular internal morphology. The CPPs with papillary internal morphology (**a**. T2WI, **b**. T1WI, **c**. post-contrast). In the pathology image of case b, the tumour cells are arranged in a papillary structure with vascular at the centre (**d**). The CPC (**e**. FLAIR) and aCPPs (**f**, **g**. post-contrast) with lobulated internal morphology. In the pathology image of case e, the tumour cells are arranged closely, with focal necrosis and haemorrhage (**h**). The CPCs with irregular internal morphology (**i**. FLAIR, **j**. T2WI, **k**. post-contrast). In the pathology image of case i, the papillary structure is irregular and complex, and the distribution of tumour cells is dense (**l**)

Internal morphology on MRI were categorized into: ① Purely cystic: no MRI visible solid lesion, but only thickened choroid plexus attaching to cysts; ② Papillary: cauliflower-like appearance with long and thin branches, arranged loosely; ③ Lobulated: petal-like appearance with short and thick branches, arranged tightly; and ④ Irregular: irregular shape or massive appearance with fuzzy/lost internal architecture on MRI. A combination of T1-weighted, T2-weighted, FLAIR sequences and T1-weighted post-contrast imaging was used to define internal morphology. The CPTs with typical papillary/lobulated/irregular internal morphology was shown in Fig. 1.

### Statistical analysis

The Spearman correlation coefficient (*r*_*s*_) was used to evaluate the associations of pathological grade with tumour longest diameter, oedema diameter, and hydrocephalus. The Mann-Whitney U test and the Kruskal-Wallis H test were applied to analyse the relationships of pathological grades to tumour locations, internal morphology, signal intensity on T1WI and T2WI, degree of enhancement, intratumoural haemorrhage, necrosis, and metastases. Statistical significance was defined as *P* < 0.05. Statistical analyses were performed using IBM SPSS statistics software, version 21.0 (IBM, Armonk, NY).

## Results

### Clinical symptoms

Patients’ symptoms included dizziness/headache (*n* = 11), asymptomatic (*n* = 7), increased head circumference (*n* = 4), seizures (*n* = 3), vomiting (*n* = 2), limb weakness (*n* = 2), ataxia (*n* = 2), poor mental condition (*n* = 1), facial numbness (*n* = 1), tinnitus (*n* = 1), and blurred vision (*n* = 1). The median duration of symptoms was 2 months, with a range of 0 months to 12 years.

### MRI findings

Among the 35 CPTs, 14 were located in the lateral ventricle (7 CPPs, 3 aCPPs, 4 CPCs), 12 in the fourth ventricle (7 CPPs, 5 aCPPs, 0 CPCs), and 3 each were found in the third ventricle (1 CPP, 2 aCPPs, 0 CPCs), cerebellopontine angle (2 CPPs, 1 aCPP, 0 CPCs), and brain parenchyma (1 CPP, 0 aCPPs, 2 CPCs). One case (CPC) located in the brain parenchyma had multiple metastasis in the cerebellopontine angle, cerebral hemisphere, cerebellum and vertebral canal.

**Fig. 2:**
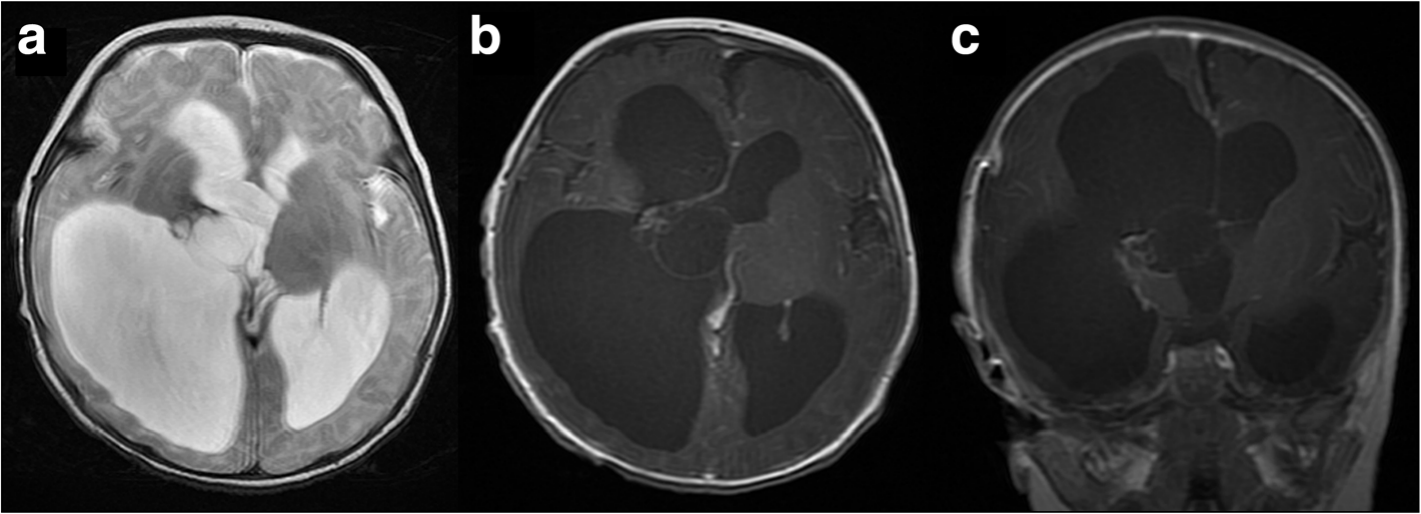
CPP (grade I). A 3-month-old boy with a purely cystic lesion in the right foramen of Monro. Bilateral lateral ventricles have severe obstructive hydrocephalus, and T2WI shows that the signal intensity in the cyst is identical to that of the CSF (**a**). Axial (**b**) and coronal (**c**) post-contrast images show that only thickened enhanced choroid plexus is attached to the cyst wall

**Fig. 3:**
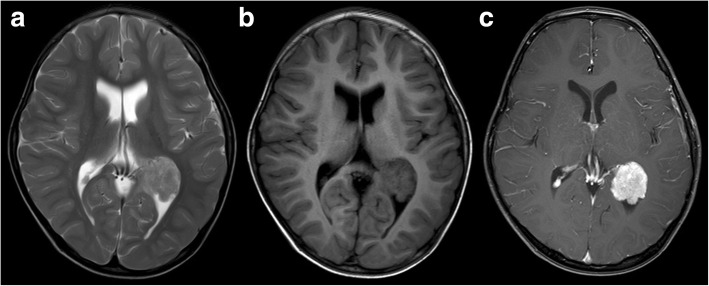
CPP (grade I). A 4-year-old boy with a papillary lesion in the left lateral ventricular trigone. The tumours are slightly hyperintense on T2WI (**a**), isointense on T1WI (**b**), and strongly enhanced in post-contrast imaging (**c**) and are connected to the choroid plexus by a vascular pedicle

CPPs (grade I): Tumours ranged from 0 to 54.9 mm in the longest diameter, with a median diameter of 28.6 mm. Among the 18 cases, 4 were purely cystic, without MRI-visible solid lesions, only thickened choroid plexus attaching to the cyst wall (Fig. 2); 9 showed papillary appearance (Fig. 3); 4 showed lobulated appearance; and 1 showed irregular appearance on MRI. The solid parts of tumours were well circumscribed, slightly hypo- (*n* = 7) or isointense (*n* = 7) on T1WI, and slightly hyper- (*n* = 10) or isointense (*n* = 4) on T2WI and FLAIR sequences. The 3 cases that underwent DWI scanning were hypo- (*n* = 1) or isointense (*n* = 2). All 14 cases with solid lesions were moderately (*n* = 5) or strongly (*n* = 9) enhanced. Eight of the 18 cases were complicated with mild (*n* = 5) or severe (*n* = 3) hydrocephalus, and severe cases showed extreme dilation of the ventricular system and obviously compressed parenchyma. Six cases were accompanied by cyst(s), and 1 case had peritumoural oedema, which was attributed to a cyst as large as 40.4 mm. Two cases revealed intratumoural haemorrhage.

**Fig. 4:**
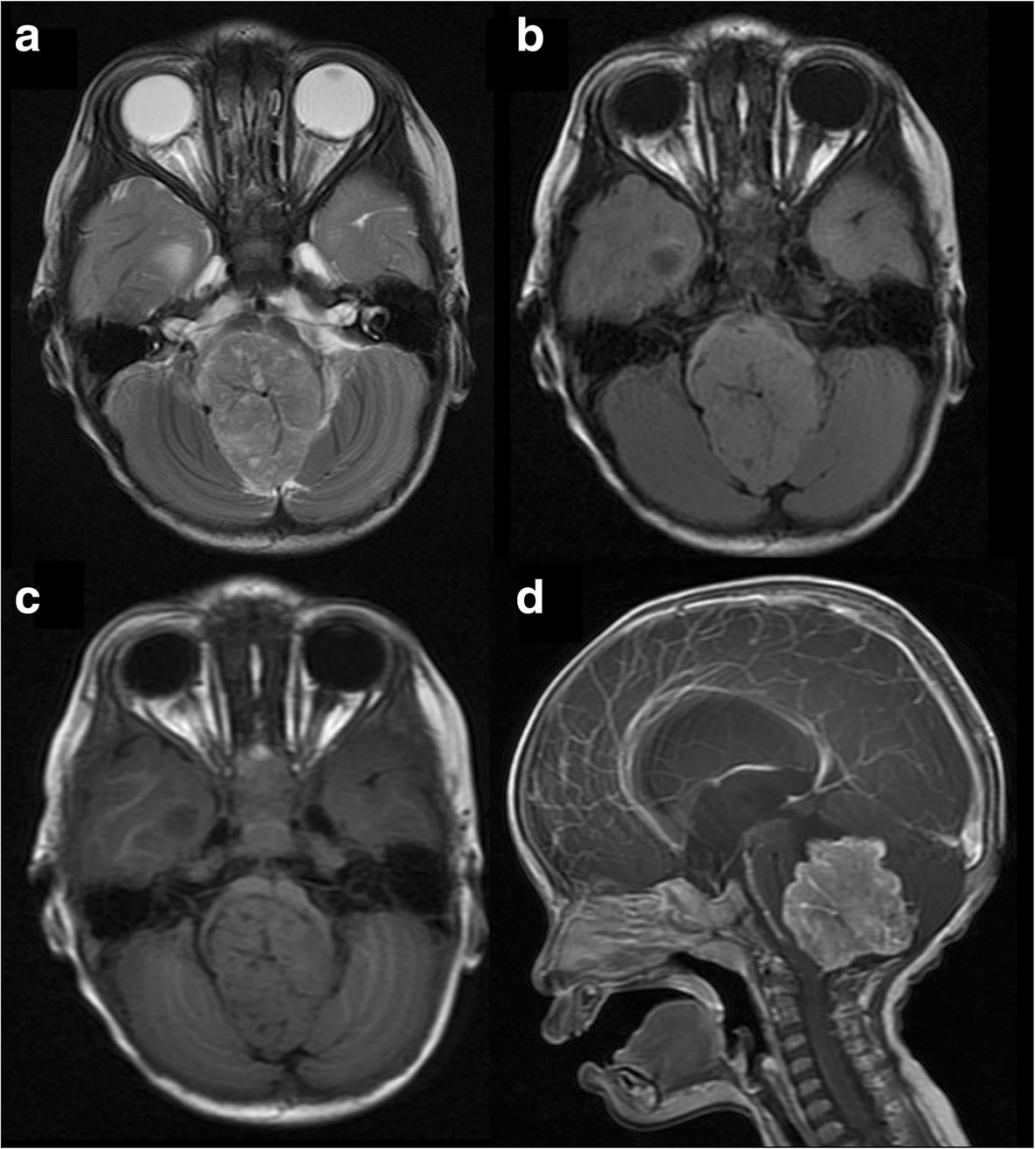
aCPP (grade II). A 2-year-old girl with a lobulated lesion in the fourth ventricle. The tumour is slightly hyperintense on T2WI (**a**) and FLAIR sequence (**b**) and isointense on T1WI (**c**), with a more heterogeneous internal structure. Sagittal post-contrast imaging shows that the tumour is strongly enhanced (**d**), and serious hydrocephalus is visible in the supratentorial ventricles

aCPPs (grade II): Tumours ranged from 19.5 to 55.8 mm in the longest diameter, with a median diameter of 44.6 mm. Among the 11 cases, 2 showed papillary appearance; 7 showed lobulated appearance (Fig. 4); and 2 had irregular appearance without papillary or lobulated architectures. The solid parts of tumours were also slightly hypo- (*n* = 5) or isointense (*n* = 6) on T1WI, slightly hyper- (*n* = 5) or iso (*n* = 4) or slightly hypointense (*n* = 2) on T2WI and FLAIR sequences, and moderately (*n* = 7) or strongly (*n* = 4) enhanced, similar to that of grade I, but the internal signal was more heterogeneous. The 3 cases that underwent DWI scanning were slightly hypo- (*n* = 1) or isointense (*n* = 2). Nine of the 11 cases were complicated with mild (*n* = 4) or severe (*n* = 5) hydrocephalus. Three cases were clinging to the ventricle wall and revealed peritumoural oedema. Four cases were accompanied by cyst(s); 4 cases had intratumoural haemorrhage; 1 case had necrosis.

**Fig. 5:**
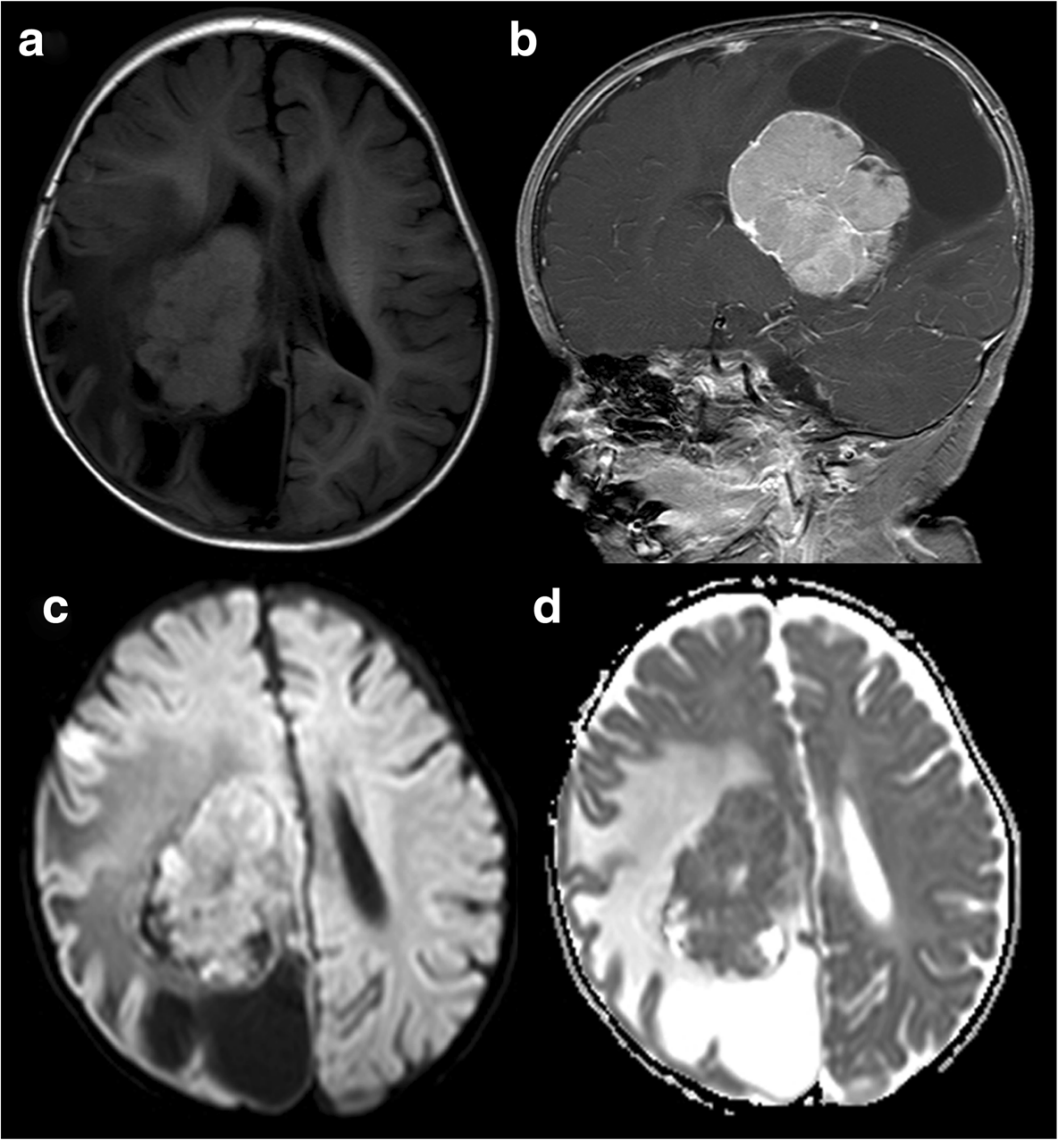
CPC (grade III). An 8-month-old girl with a lobulated lesion in the right lateral ventricular trigone. The solid part of the tumour is isointense on T1WI with multiple intratumoural necrosis and cysts. The surrounding brain parenchyma shows widely arranged oedema (**a**). Sagittal post-contrast imaging shows that the tumour is strongly enhanced (**b**). DWI sequence (**c**) reveals a hyperintense area, corresponding with a slightly hypointense area on the ADC map (**d**)

**Fig. 6:**
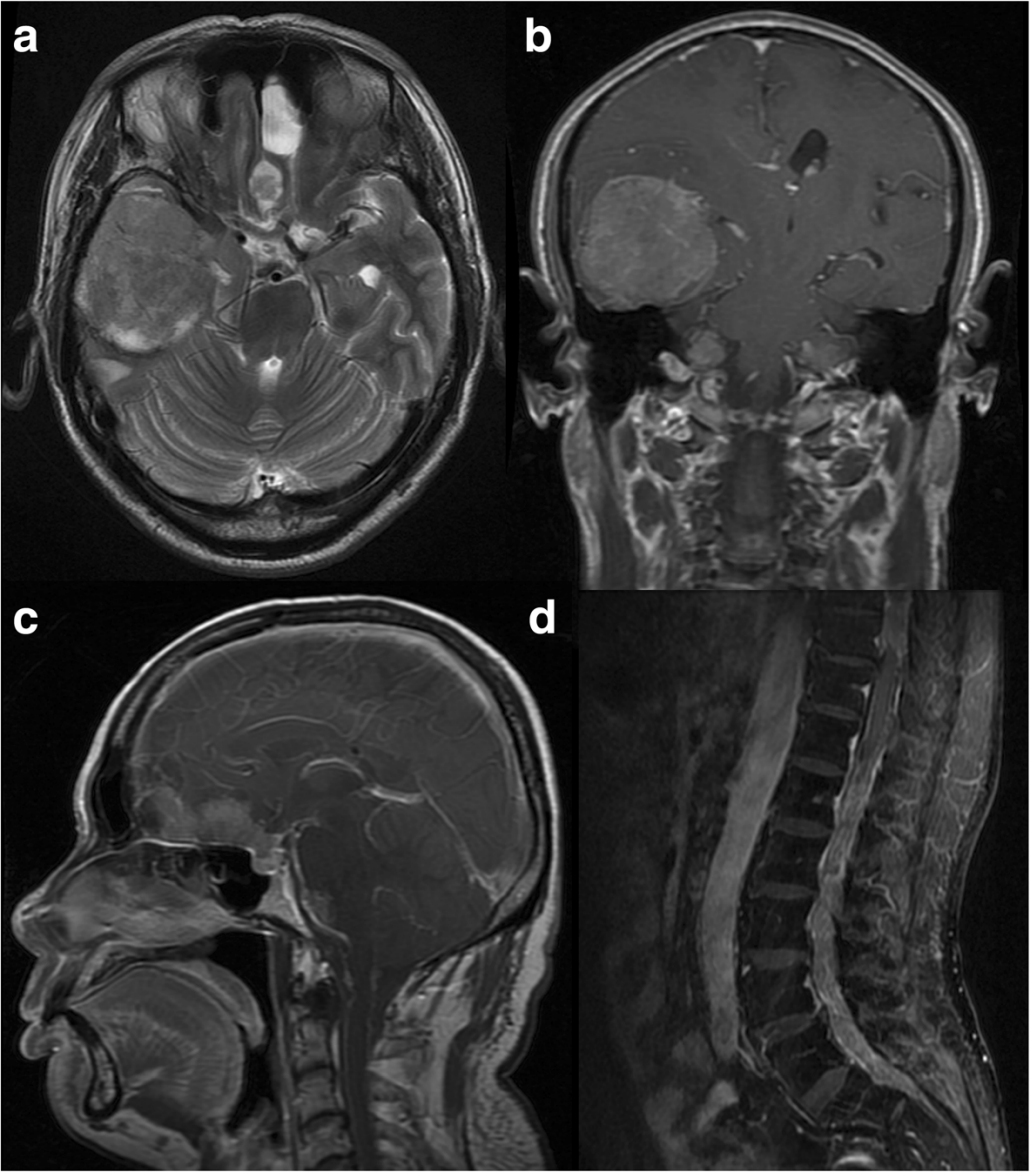
CPC (grade III). A 59-year-old male with an irregular massive lesion in the right temporal lobe, with multiple intracranial solid-cystic metastases. The solid part of the tumour is heterogeneous hyperintense on T2WI (**a**) and moderately enhanced in post-contrast imaging (**b**). Four years after the gross resection of the temporal lobe lesion, the solid-cystic metastases are increased and enlarged (**c**). Disseminations are shown in the spinal canal and are strongly enhanced in the post-contrast imaging (**d**)

CPCs (grade III): Tumours ranged from 55.7 to 81.3 mm in the longest diameter, with a median diameter of 60.6 mm. Among the 6 cases, 1 showed papillary appearance; 3 showed lobulated appearance (Fig. 5); and 2 had irregular or massive appearance (Fig. 6). The solid parts of tumours were slightly hypo- (*n* = 2) or isointense (*n* = 4) signal intensity on T1WI, slightly hyper- (*n* = 5) or isointense (*n* = 1) on T2WI and FLAIR sequences, and moderately (*n* = 4) or strongly (*n* = 2) enhanced. Tumour internal signals were heterogeneous. The 2 cases that underwent DWI scanning were essentially isointense, but 1 had a high-intensity area, corresponding with slightly low intensity on the ADC map. Three of the 6 cases were complicated with mild (*n* = 2) or severe (*n* = 1) hydrocephalus. All of the 6 cases significantly compressed the ventricle wall with widely arranged peritumoural oedema. Two cases were accompanied by cyst(s); 1 case revealed intratumoural haemorrhage; 2 case revealed necrosis; and 1 case had multiple intracranial metastases and had disseminated into the spinal canal.

### The MRI features associated with the pathological grades

**Fig. 7:**
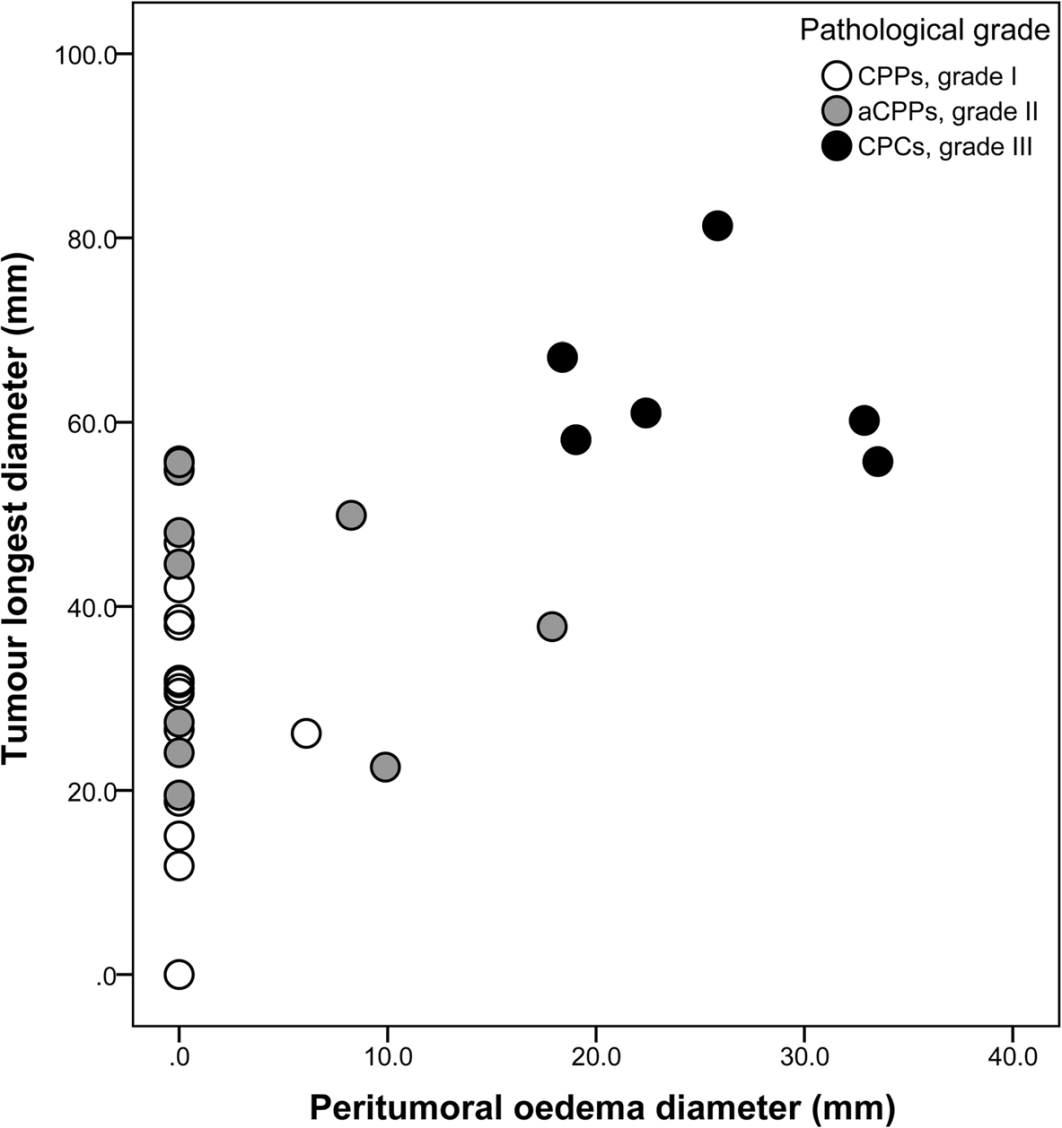
Distribution of tumour longest diameter and oedema diameter in relation to pathological grades

Spearman correlation analysis showed that the pathological grades of CPTs were statistically associated with tumour longest diameter (*r*_*s*_ = 0.68, *P* < 0.001) and oedema diameter (*r*_*s*_ = 0.72, *P* < 0.001) but were not related to the degree of hydrocephalus (*r*_*s*_ = 0.19, *P* = 0.277) (Table 1). All 6 cases with CPCs had both large tumours over 55 mm in the longest diameter and a large amount of peritumoural oedema, while the other cases did not have these characteristics (Fig. 7).

**Table 1:** MRI Findings Associated with the Pathological Grades in 35 CPTs

MRI features	Pathological grades			*P*
	I	II	III	(*r*_*s*_)
Tumour longest diameter (mm)	28.6	44.6	60.6	< 0.001
median (min, max)	(0, 54.9)	(19.5, 55.8)	(55.7, 81.3)	(0.68)
Internal morphology				
purely cystic	4	0	0	
papillary	9	2	1	0.016
lobulated	4	7	3	
irregular	1	2	2	
Necrosis				
no	18	10	4	0.023
yes	0	1	2	
Oedema diameter (mm)	0	0	24.1	< 0.001
median (min, max)	(0, 6.1)	(0, 17.9)	(18.4, 33.5)	(0.72)

Mann-Whitney U test indicated that the pathological grade of CPTs was statistically associated with peritumoural oedema (*Z* = 3.87, *P* < 0.001) and necrosis (*Z* = 2.27, *P* = 0.023) but was not related to tumour locations (*Z* = 0.46, *P* = 0.648), signal intensity (slightly hypo-, isointense) on T1WI (*Z* = 0.62, *P* = 0.534), degree of enhancement (*Z* = 1.52, *P* = 0.130), intratumoural haemorrhage (*Z* = 0.97, *P* = 0.330), and metastases (*Z* = 1.58, *P* = 0.115) (Table 1).

Kruskal-Wallis H test revealed that the pathological grade of CPTs was statistically associated with internal morphology (*χ*^2^ = 10.32, *P* = 0.016) but not to signal intensity (slightly hyper-, hypo-, isointense) on T2WI (*χ*^2^ = 0.50, *P* = 0.777) (Table 1).

## Discussion

### MRI similarities in CPTs of different grades

CPTs can be found in patients of all ages, but as many as 70–80% occur in children, comprising 10–20% of brain tumours within the first year of life [2]. In our study, 66% (23/35) of patients were under the age of 18, and 26% (9/35) of patients were below 1 year old, which was generally in accordance with previous studies. CPTs originate from the choroid plexus epithelium and arise anywhere this tissue existed [5]. Based on the amount of choroidal tissue present, CPTs are generally found within the ventricular system [1, 2], in which the most common site is the lateral ventricle, followed by the fourth ventricle and the third ventricle [5]. In our study, 83% (29/35) of cases were located in the ventricular system, including 40% in the lateral ventricle, 34% in the fourth ventricle, and 9% in the third ventricle, consistent with previous studies. On MRI, some cases could be found connected to the choroid plexus by a vascular pedicle. There were 17% of cases in our study located outside the ventricular system, with 3 each in the cerebellopontine angle and the brain parenchyma. Along with the rare locations, such as the suprasellar, pineal gland, cerebellum and spinal epidural [2], these extraventricular CPTs are speculated to be an embryonic remnant of the choroid plexus [5].

It has been reported that signal characteristics and enhancement patterns cannot distinguish between benign and malignant CPTs [9]. Regardless of pathological grade, the CPTs in our study were generally slightly hypo- or isointense on T1WI, slightly hyper- or isointense on T2WI, and moderately or strongly enhanced in post-contrast imaging. Heterogeneity depended on the amounts of calcification, haemorrhage and necrosis. Slightly hypointense on T2WI was not commonly seen in CPTs unless the areas of calcification or chronic haemorrhage were large and dense. A CT or SWI sequence scan would be required to distinguish these probabilities.

CPTs have long been associated with hydrocephalus and symptoms related to increased intracranial pressure [5, 10]. In our study, degrees of hydrocephalus were revealed in 57% (20/35) of cases. Hydrocephalus in CPTs is a combined result of obstructive hydrocephalus, increased CSF production, and impaired CSF reabsorption at the arachnoid granulations [2]; thus, the degree of hydrocephalus is not always proportional to tumour size, distinct from other intraventricular tumours. One or more cysts were revealed in 34% (12/35) of the cases in our study, with the mural nodule or thickened choroid plexus attaching inside or outside the cyst wall. Our pathological reports called this observation “CPT accompanying arachnoid cyst”, but some previous studies called it “cystic CPTs” and considered that the cyst content was identical to CSF [11]. We presumed that the cysts might be a result of arachnoid adhesion and CSF production by the tumour.

### MRI distinctions among CPTs of different grades

A preoperative diagnosis of CPT is very useful, even if surgery is performed for all cases. It may allow the surgeon to adapt the surgical strategy (possible preoperative embolization for example) because CPCs are more invasive and tend to be more haemorrhagic [8]. Therefore, we tried to explore a grading strategy for CPTs using MRI.

Histologically, CPPs resemble the non-neoplastic choroid plexus with higher cellular density, while aCPPs exhibit increased mitotic activity, and CPCs show various histological features of malignancy, including very high cellular density, pleomorphism, reduction of the papillary pattern, necrosis, and invasion of adjacent parenchyma [1, 8, 12, 13]. These histologic trends are in agreement with what is shown on MRI. Our study indicated that tumour longest diameter, papillary appearances, necrosis and peritumoural oedema diameter were relevant to the pathological grade based on the MRI findings from 35 CPTs.

CPCs were generally larger than CPPs [2], but the specific diameter of each grade of CPTs had not been previously reported in the literature. In our study, the median tumour largest diameters for each grade were 28.6, 44.6, and 60.6 mm, respectively, which were positively correlated to the pathological grade (*r*_*s*_ = 0.68). Brisk mitotic activity is one of the features of malignancy [1]. We presumed that the aggressive growth pattern might attribute to larger tumour volume.

Frequent invasion of the brain parenchyma is one of the signs of malignancy in CPTs [1]. A previous study stated that aCPPs occasionally grew into the adjacent white matter, while CPCs always grew into the brain parenchyma through the ventricle wall, and the invasive margins could appear as associated vasogenic oedema [2, 14]. Peritumoural oedema presented in 100% (6/6) of CPCs in our study, while it only occurred in 6% (1/18) of CPPs and 27% (3/11) of aCPPs. In pathology, all CPCs and 1 aCPP with peritumoural oedema had adjacent brain invasion. The median oedema diameter in CPCs was as large as 24.1 mm, while in aCPPs and CPCs, the medians were only 0 mm. The sizes of peritumoural oedema were significantly different between papillomas (CPP, aCPP) and CPCs, and the size was positively correlated with the pathological grade of CPTs (*r*_*s*_ = 0.72) according to the statistical analysis.

The combination of tumour longest diameter and oedema diameter was helpful in predicting the grade of CPTs by MRI. As is shown in Fig. 7, all CPCs were over 55 mm, with a large scale of peritumoural oedema, while the only aCPPs over 55 mm presented no oedema. The only CPP with oedema was only 26.2 mm in the solid portion, but the venous compression by multiple cysts had resulted in oedema.

CPTs are always described as lobulated [13] or cauliflower [2, 15] in appearance on MRI. Our study was the first to categorize CPTs into papillary, lobulated and irregular according to the MRI appearance of their solid portion, which represented the reduction of branches and the increase in solid area. This categorizing method was based on histology. CPPs feature a fibrovascular stalk surrounded by a single layer of cuboidal to columnar epithelium arranged in a papillary configuration [2]. The papillary architecture blur with increasing malignancy, while in CPCs, the papillary pattern decreased and is replaced with solid areas [12, 15, 16]. Despite the fact that tumours with solid portions that could be categorized by internal morphology, 4 cases were purely cystic, with thickened choroid plexus attaching to the cyst wall. These 4 cases were classified into a separate category and were all CPPs. Purely cystic CPT has been reported as a very rare pathological entity [17], and all cases described thus far have been of CPPs [11], which was consistent with our study. Although purely cystic CPTs are always benign in histology, they can cause severe obstructive hydrocephalus that can lead to sudden death [17]. Thus, the awareness of this special form of CPT should be increased.

## Limitations

Our study had 2 limitations. First, the sample size was small. Although 35 is not a small sample size for CPTs, the power for hypothesis testing might be decreased, especially for factors with low incidence rates. For example, only 1 case presented metastasis, which made it hard to predict the pathological grade with this factor, even though it indicated malignancy in histology. With the accumulation of cases, more factors relevant to the grade of CPTs might be verified.

Second, only conventional MRI findings were taken into account. DWI was always used as a tool to grade intracranial tumours, but only 8 cases (3 CPPs, 3 aCPPs, 2CPPs) in our study had DWI data. All of the 8 cases were slightly hypo- or isointense, despite 1 CPC with local high signal, which might indicate a region of higher malignancy. The value of DWI in grading CPTs remained to be confirmed. Arterial spin-labelling (ASL) perfusion MRI can reflex blood flow conditions without contrast agent. It has been reported that CPCs tend to have higher numbers of micro vessels and very dissimilar organization, and ASL can differentiate between CPCs and papillomas (CPPs, aCPPs) according to relative cerebral blood flow (CBF) value [8]. In a following study, we will add more new sequences such as DWI and ASL to the cases with suspected CPT to provide more information for grading diagnosis.

## Conclusion

CPTs typically appear as intraventricular papillary or lobulated lesions on MRI. They are generally moderately or strongly enhanced and are often accompanied by degrees of hydrocephalus. Larger tumour volume, irregular or fuzzy internal morphology, presentation of necrosis and wide-ranging peritumoural oedema may increase the likelihood of malignancy. These findings shall help us to improve the pretreatment diagnosis and provide more guidance for clinical decisions.

## Abbreviations

aCPP: Atypical choroid plexus papilloma
CPC: Choroid plexus carcinoma
CPP: Choroid plexus papilloma
CPT: Choroid plexus tumour
